# Associations between Unconventional Natural Gas Development and Nasal and Sinus, Migraine Headache, and Fatigue Symptoms in Pennsylvania

**DOI:** 10.1289/EHP281

**Published:** 2016-08-25

**Authors:** Aaron W. Tustin, Annemarie G. Hirsch, Sara G. Rasmussen, Joan A. Casey, Karen Bandeen-Roche, Brian S. Schwartz

**Affiliations:** 1 Department of Environmental Health Sciences, Johns Hopkins Bloomberg School of Public Health, Baltimore, Maryland, USA; 2 Center for Health Research, Geisinger Health System, Danville, Pennsylvania, USA; 3 Robert Wood Johnson Health and Society Scholars Program, University of California, San Francisco, San Francisco and University of California, Berkeley, Berkeley, California, USA; 4 Department of Biostatistics, Johns Hopkins Bloomberg School of Public Health, Baltimore, Maryland, USA; 5 Department of Medicine, Johns Hopkins School of Medicine, Baltimore, Maryland, USA

## Abstract

**Background:**

Unconventional natural gas development (UNGD) produces environmental contaminants and psychosocial stressors. Despite these concerns, few studies have evaluated the health effects of UNGD.

**Objectives:**

We investigated associations between UNGD activity and symptoms in a cross-sectional study in Pennsylvania.

**Methods:**

We mailed a self-administered questionnaire to 23,700 adult patients of the Geisinger Clinic. Using standardized and validated questionnaire items, we identified respondents with chronic rhinosinusitis (CRS), migraine headache, and fatigue symptoms. We created a summary UNGD activity metric that incorporated well phase, location, total depth, daily gas production and inverse distance–squared to patient residences. We used logistic regression, weighted for sampling and response rates, to assess associations between quartiles of UNGD activity and outcomes, both alone and in combination.

**Results:**

The response rate was 33%. Of 7,785 study participants, 1,850 (24%) had current CRS symptoms, 1,765 (23%) had migraine headache, and 1,930 (25%) had higher levels of fatigue. Among individuals who met criteria for two or more outcomes, adjusted odds ratios for the highest quartile of UNGD activity compared with the lowest were [OR (95% CI)] 1.49 (0.78, 2.85) for CRS plus migraine, 1.88 (1.08, 3.25) for CRS plus fatigue, 1.95 (1.18, 3.21) for migraine plus fatigue, and 1.84 (1.08, 3.14) for all three outcomes together. Significant associations were also present in some models of single outcomes.

**Conclusions:**

This study provides evidence that UNGD is associated with nasal and sinus, migraine headache, and fatigue symptoms in a general population representative sample.

**Citation:**

Tustin AW, Hirsch AG, Rasmussen SG, Casey JA, Bandeen-Roche K, Schwartz BS. 2017. Associations between unconventional natural gas development and nasal and sinus, migraine headache, and fatigue symptoms in Pennsylvania. Environ Health Perspect 125:189–197; http://dx.doi.org/10.1289/EHP281

## Introduction

Unconventional natural gas development (UNGD), which includes the process of hydraulic fracturing, represents an expanding share of energy production worldwide. Shale gas extraction now comprises 40% of U.S. domestic natural gas production [Energy Information Administration (EIA 2015)]. In the past decade, particularly rapid increases in UNGD have occurred in Pennsylvania, where > 8,800 unconventional wells have been drilled.

There are concerns that UNGD could affect the environment via chemical pollutants such as diesel exhaust, volatile organic compounds, combustion products, fugitive emissions, and fracking chemicals (Werner et al. 2015). UNGD has been linked to contamination of air (Macey et al. 2014; Paulik et al. 2015), soil (Maloney and Yoxtheimer 2012), ground water (Jackson et al. 2013; Drollette et al. 2015), and surface water (Kassotis et al. 2014). UNGD also creates contextual and psychosocial stressors including noise, truck traffic, influxes of nonlocal workers, and perceived negative impacts on quality of life and on the built and social environments (Saberi et al. 2014; Powers et al. 2015; Adgate et al. 2014).

There have been few studies of the health effects of UNGD, despite increasing concerns (Mitka 2012; Kovats et al. 2014). Previous studies have been limited by factors including small sample size and imprecise exposure assessment (Adgate et al. 2014). Because the expansion of UNGD has outpaced scientific understanding of its potential health impacts, studies of self-reported outcomes have been advocated as a rapid means of generating hypotheses that could influence public policy. Furthermore, some illnesses with plausible links to UNGD, such as pain syndromes and fatigue, are defined solely by symptoms. Yet, to date there have been only two epidemiologic studies of symptoms in relation to UNGD, each with < 500 participants (Steinzor et al. 2013; Rabinowitz et al. 2015).

We used data from a large population-based cross-sectional survey of Pennsylvania adults to identify patients with nasal and sinus symptoms, migraine headache, and higher levels of fatigue. We selected these outcomes because of their high prevalence, large economic costs, and possible links to environmental risk factors through chemical toxicity, irritation, odors, or stress (Hastan et al. 2011; Bhattacharyya 2009; Shashy et al. 2004; Tan et al. 2013; Friedman and De ver Dye 2009; Sjöstrand et al. 2010; Bell et al. 1998; Griffith and Zarrouf 2008; Ranjith 2005; Ricci et al. 2007). The purpose of this study was to test the null hypothesis that UNGD is not associated with these three outcomes. To do so, we performed a case–control analysis in which we compared individuals having one or more of these health outcomes with selected participants having no or minimal evidence of these diseases.

## Methods

### Study Overview

In early 2014, we performed a cross-sectional survey of primary care patients of the Geisinger Clinic. Information was gathered via a questionnaire designed to study general chronic rhinosinusitis (CRS) epidemiology (for the questionnaire, see Supplemental Material, “Population Study of Nasal and Sinus Symptoms”). The questionnaire did not mention UNGD because that was not its primary purpose. We used residential addresses and information about Pennsylvania unconventional gas wells to create UNGD activity metrics for four time-varying well development phases. We evaluated the associations between UNGD activity and CRS, migraine headache, and fatigue symptoms. The study protocol was approved by the Institutional Review Board (IRB) of the Geisinger Health System with an IRB Authorization Agreement with the Johns Hopkins Bloomberg School of Public Health. Waivers of Health Insurance Portability and Accountability Act of 1996 (HIPAA) authorization and written informed consent were approved by the IRB; implied consent was considered to have been provided if the patient returned the mailed questionnaire.

### Study Population

The Geisinger Clinic provides primary care services to > 400,000 patients, predominantly in central and northeastern Pennsylvania. Our source population consisted of 200,769 adult (age ≥ 18 years) Geisinger primary care patients for whom we had electronic health record (EHR) data and information on race/ethnicity. From this source population, we selected 23,700 survey recipients using a stratified sampling design that is described in “Rationale and Description of the Stratified Sampling Method.” We mailed the baseline questionnaire in April 2014. A total of 7,847 (33.1%) individuals returned the questionnaire after three mailings. Questionnaires were returned between 13 April and 13 October 2014. After excluding respondents who lived outside Pennsylvania (*n* = 62), the study sample consisted of 7,785 participants.

### Rationale and Description of the Stratified Sampling Method

We oversampled racial/ethnic minorities because a primary interest of the parent grant was to understand racial/ethnic differences in CRS epidemiology. Geisinger’s catchment area only has ~8% racial/ethnic minorities. Oversampling was necessary to ensure a sufficient number of racial/ethnic minorities in the parent study.

Similarly, to ensure an adequate number of CRS patients in the parent CRS study, we oversampled individuals with higher likelihood of having CRS. To do so, we used EHR data to identify Geisinger primary care patients with higher, intermediate, and lower likelihood of CRS. These assessments were based on *International Classification of Diseases, Ninth Revision* (ICD-9) codes and Current Procedural Terminology (CPT) codes from the medical record. Patients with a “higher” likelihood of CRS (*n* = 13,494) had at least two ICD-9 codes for CRS (ICD-9 codes 473.x or 471.x) associated with an outpatient, inpatient, or emergency department encounter, or they had at least one CPT code for sinus computerized tomography, sinus endoscopy, or sinus surgery. Patients with “intermediate” likelihood of CRS (*n* = 49,918) had at least one ICD-9 code for asthma (493.x) or allergic rhinitis (477.x) or a single ICD-9 code for CRS associated with an outpatient, inpatient, or emergency department encounter. The 137,357 patients who did not meet criteria for the higher and intermediate likelihood groups were designated as having a “lower” likelihood of CRS.

We divided our source population into six strata based on race/ethnicity and likelihood of CRS. We mailed the baseline CRS survey to a larger percentage of individuals in the strata of interest (see Table S1).

### Covariates

We obtained the following covariates from the EHR: sex, current age (years), race/ethnicity (white non-Hispanic, other), smoking status (never, current, former), body mass index (BMI; kg/m^2^), residential address, and history of receiving Medical Assistance, a means-tested health insurance program that we used as a surrogate for family socioeconomic status (Casey et al. 2013). We used information in the EHR to derive each individual’s residential place type (township, borough, or census tract in cities) and Charlson comorbidity index. We computed the Charlson index, which incorporates the number and severity of comorbid illnesses, in a manner consistent with previously published criteria (Charlson et al. 1987). We dichotomized race/ethnicity because only 10% of participants were nonwhite, which is reflective of the general population in these communities (Casey et al. 2016). Our questionnaire ascertained additional information on educational status, marital status, household income, hay fever, nasal polyps, age at onset of nasal/sinus symptoms (in 5-year categories), history of sinus surgery, and current use of sinusitis medications (antibiotics and oral, inhaled, and nasal corticosteroids). We used U.S. Census data (Liu et al. 2012) to derive community socioeconomic deprivation (CSD) in townships, boroughs, and cities using a modified version of the Townsend index (Townsend 1987) as previously reported (Liu et al. 2012).

### Outcome Ascertainment

The cardinal symptoms of CRS are nasal congestion/obstruction, nasal discharge (anterior or posterior nasal drip), smell loss, and facial pain or pressure. Our questionnaire ascertained the frequency (“never,” “once in a while,” “some of the time,” “most of the time,” or “all the time”), in the past 3 months, of the aforementioned symptoms (questions 10–15 of the questionnaire, which is included in the Supplemental Material, “Population Study of Nasal and Sinus Symptoms”). Following European Position Paper on Rhinosinusitis and Nasal Polyps (EPOS) diagnostic criteria for CRS in epidemiologic studies (Fokkens et al. 2012), we determined participants to have current CRS if they experienced two or more cardinal symptoms [one of which must be nasal congestion/obstruction (question 10) or discharge (question 11 and/or 12)] at least “most of the time” in the past 3 months.

We ascertained migraine headache via questions from the ID Migraine™ questionnaire (Lipton et al. 2003) covering the past 12 months. Those with headaches at least “some of the time” (question 80) were asked the frequency (“never,” “rarely,” “less than half the time,” “half the time or more”) of headache-associated disability, nausea, and photophobia (questions 81–83). Using a validated scoring method (Lipton et al. 2003), we dichotomized the three responses. Responses of “never” or “rarely” were scored as no and responses of “less than half the time” or “half the time or more” were scored as yes. Participants who answered yes to at least two of three questions were considered to have migraines.

We ascertained fatigue with eight questions from the Patient-Reported Outcomes Measurement Information System (PROMIS®) fatigue short form 8a (http://www.assessmentcenter.net). These items assessed the frequency (“not at all,” “a little bit,” “somewhat,” “quite a bit,” “very much”) of fatigue and fatigue-related disability in the past week (questions 84–91). We used the instrument’s standardized scoring instructions to code responses from 1 (“not at all”) to 5 (“very much”) and summed the eight values to produce a score ranging from 8 to 40. We excluded individuals who answered fewer than four questions (*n* = 76). Individuals who answered between 4 and 7 questions were assigned a prorated score using the following formula: score = (raw sum × 8)/(number of items answered). Fractional scores were rounded up to the nearest integer. Our “higher levels of fatigue” outcome consisted of individuals in the highest quartile (score ≥ 28).

Some respondents met criteria for more than one outcome. In the analysis, we evaluated associations of UNGD with single outcomes (i.e., CRS only, migraine only, or fatigue only) and with multiple outcomes (i.e., participants with CRS and migraine, CRS and fatigue, migraine and fatigue, or all three outcomes).

### Reference Group

We performed an unmatched case–control analysis in which we compared individuals having one or more of the three primary outcomes (“cases”) with a subset of participants having no or minimal evidence of these outcomes (hereafter referred to as “controls” or the “reference group”). The reference group comprised study participants who *a*) did not meet diagnostic criteria for past or current CRS, *b*) reported no migraine headache symptoms, and *c*) reported lower levels of fatigue (i.e., first quartile of fatigue score). Individuals with past CRS, intermediate likelihood of migraine, and/or moderate levels of fatigue were excluded from the reference group. These exclusion criteria were intended to produce a reference group free of individuals with a moderate likelihood of having the outcome (in the case of migraine and fatigue) or whose disease had been aggressively managed and treated (in the case of past CRS).

We created the reference group as follows. First, we excluded all study participants with one or more of the outcomes of interest. Next, individuals who met criteria for lifetime CRS [i.e., responses of “yes” to at least two cardinal symptoms on questions 1–6, one of which had to be nasal blockage (question 1) or discharge (question 2 and/or 3)] but not current CRS were deemed to have “past CRS” and were excluded from the reference group. We then excluded participants from the reference group if they endorsed any of the three ID Migraine™ criteria. In other words, members of the reference group either skipped the ID Migraine™ questions (e.g., because they reported a headache frequency of “never” or “once in a while” on question 80 and were instructed to skip the following three questions) or responded to questions 81–83 with no migraine symptom occurring more frequently than “never” or “rarely.” Finally, we excluded individuals from the reference group if their fatigue score was higher than the 25th percentile (i.e., those with fatigue score > 13) or if they did not answer at least four of eight PROMIS® fatigue items (questions 84–91). No other inclusion or exclusion criteria were applied to the reference group.

### UNGD Activity Assessment

We used published descriptions, and our own data, to estimate the duration of each UNGD phase (Gaines and Ziegler 2013; New York State Department of Environmental Conservation 2015; Casey et al. 2016). Pad preparation, which involves clearing of the well site, lasts ~30 days. Drilling the well then takes 1–30 days, proportionate to the total (vertical plus horizontal) depth. After drilling, hydraulic fracturing (fracking) occurs during a stimulation phase that lasts an average of 7 days. Finally, the well produces natural gas during a production phase that lasts months to years.

To capture these complexities of well development, we compiled data on UNGD in Pennsylvania from 1 January 2005 through 31 December 2014 from the Pennsylvania Department of Environmental Protection, the Pennsylvania Department of Conservation and Natural Resources, and SkyTruth (http://skytruth.org). We obtained the following information for each well: geographic coordinates; start dates of drilling, stimulation, and production; total depth; and volume of natural gas produced during 6- or 12-month reporting windows.

Using methods described previously (Casey et al. 2016), we created UNGD activity metrics for each phase of well development. Briefly, these metrics incorporated all unconventional gas wells in Pennsylvania and were defined as follows:


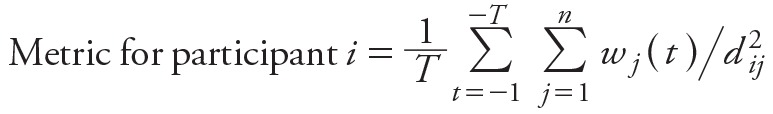
_,_

where *T* is an averaging period in days (in our primary analysis, *T* = 90 because CRS diagnostic criteria require 3 months of symptoms); *t* is a temporal summation index whose negative sign represents past dates (e.g., summing from *t* = –1 to –90 indicates that the metric was averaged over 90 consecutive days immediately before the survey); *n* is the number of wells; *w_j_*(*t*) is the weight assigned to the *j*th well on day *t*; and *d* ^2^ *_ij_* is the squared distance between well *j* and the residential address of participant *i*. We set *w_j_*(*t*) = 0 for wells that were inactive in the given phase on day *t*. Active wells were assigned weights during the duration of the relevant phase as follows: for pad preparation and drilling metrics, *w_j_*(*t*) was 1; for the stimulation metric, *w_j_*(*t*) was the total depth in feet (a surrogate for hydraulic fracturing chemical volumes and the number of truck trips required to transport stimulation materials); and for the production metric, *w_j_*(*t*) was the average daily volume in Mcf (1 Mcf = 1,000 cubic feet) of natural gas produced during the corresponding reporting period.

Because the four UNGD phase metrics were highly correlated when averaged over 90 days (Spearman coefficient > 0.90 for each pairwise comparison), we *z*-transformed the metrics and summed the resulting *z*-scores. For analysis, we divided this continuous composite UNGD activity metric into quartiles for ease of interpretation and because of its skewed distribution.

### Statistical Analysis

We used descriptive statistics to compare characteristics of participants with and without each outcome. To evaluate selection bias with respect to UNGD, we compared distributions of the UNGD activity metric in study participants and questionnaire nonresponders. To assess the potential for nonconservative errors due to selection bias with respect to health status, we analyzed distributions of the Charlson comorbidity index in study participants and survey nonresponders, stratified by UNGD quartile. Categorical and continuous variables were compared using χ^2^ tests and *t*-tests, respectively. For hypothesis testing, *p*-values < 0.05 were considered statistically significant.

We used weighted logistic regression to evaluate associations between UNGD activity and symptoms while adjusting for confounding variables. All models compared individuals with the outcome(s) of interest (“cases”) to the reference group described above (“controls”). The use of sampling weights allowed us to account for the differential patient selection and participation rates in our stratified design while targeting unbiased measures of association and obtaining robust standard errors. We assigned each participant a sampling weight equal to the inverse probability of inclusion in the study (see Table S1). Because the weight in one stratum (150.8) was substantially larger than the other weights, we truncated this weight by reducing it to the value of the second-highest weight (32.3).

We adjusted all models for these potential confounders that we identified *a priori*: sex, race/ethnicity (non-Hispanic white vs. other), age [linear and quadratic terms; to avoid collinearity, we centered the age variable by subtracting its mean (i.e., *A_c_* = *A_i_* – *A*_mean_)], receipt of Medical Assistance (never vs. ever), and smoking status (never vs. former and current). We tested for additional confounding by adding linear and quadratic terms for BMI and CSD. We retained these covariates in the models if they changed associations between UNGD and the outcome by ≥ 10%. Analyses were performed in R (version 3.0.2, R Project for Statistical Computing) and Stata 13.1 (StataCorp) using the svy command.

We reasoned that UNGD might be associated with current CRS only for onset of symptoms after 2006, when UNGD commenced in Pennsylvania. To test the associated hypothesis, we stratified the CRS group by date of symptom onset (before/after 1 January 2006) and reran models within each stratum. Although associations of UNGD activity with our other outcomes could also differ by onset date, our questionnaire did not ascertain the onset date of migraine and fatigue symptoms.

We performed several sensitivity analyses. To explore the impact of sampling weight choices, we reran models with full (i.e., not truncated) weights and again with no weights. To determine whether associations differed by the length of the UNGD assessment period, we compared associations using 7-day, 90-day, and 365-day averaged UNGD metrics that corresponded to the questionnaire’s recall windows for the three primary outcomes. To explore spatial differences among groups of participants, we mapped the residential locations of individuals with and without our primary outcomes stratified by UNGD quartile and case/control status. To assess whether UNGD was associated with symptoms in individuals with past disease or moderate symptoms, we created additional CRS and fatigue models in which we reclassified some previously excluded individuals as “cases” (for details see Supplemental Material, “Models of Past Disease and Moderate Symptoms”). To assess whether unmeasured confounding, including spatial confounding, could be responsible for the observed associations, we created “negative control outcome” models (Lipsitch et al. 2010). These adjusted logistic regression models evaluated associations between UNGD and self-reported outcomes (bad breath, ear pain, and cold/flu symptoms) that we thought were unlikely to be related to UNGD. We expected to find no significant associations between UNGD and these outcomes; the presence of such associations could indicate bias resulting from unmeasured confounding. In these models, we defined cases as all study participants who reported the symptom at least “most of the time” in the past 3 months (questions 36, 43, and 48 for bad breath, ear pain, and cold/flu symptoms, respectively). The reference group for each model consisted of all individuals who reported the symptom “never” in the past 3 months.

## Results

### Characteristics of the Study Population

**Figure 1:**
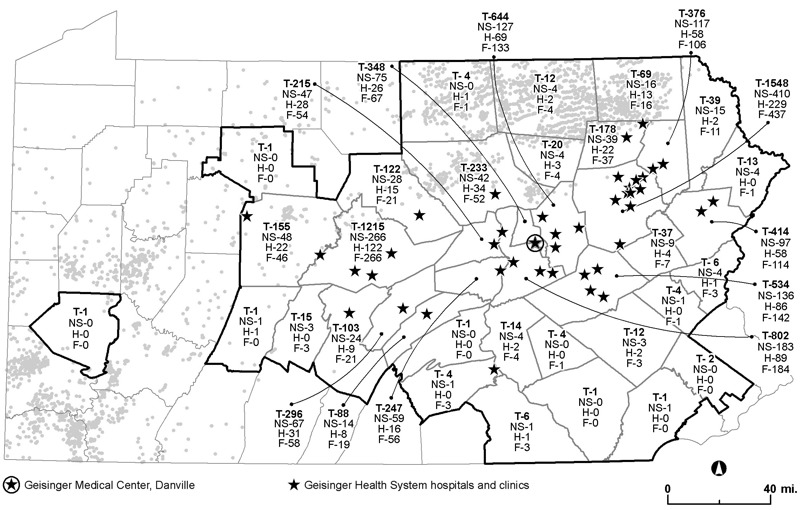
Map of study area. Thick black outlines designate Pennsylvania counties with at least one participant [from U.S. Census Bureau TIGER/line files (U.S. Census Bureau 2010)]. Numbers within the borders of each county indicate the total number of participants (T) and the number with chronic rhinosinusitis symptoms (NS), migraine headache (H), and higher levels of fatigue (F) (data from the Geisinger Clinic). Gray circles show locations of drilled unconventional natural gas wells as of December 2014 (Pennsylvania Department of Environmental Protection 2016). Black stars represent Geisinger hospitals and clinics. Map was made with ArcGIS Desktop (release 10, Esri, Redlands, CA).

Questionnaire respondents were 7,785 individuals from 39 counties in central and northeastern Pennsylvania, in regions with and without UNGD (Figure 1). Compared with questionnaire recipients who did not respond, our study population was more likely to be female, white, and older (results not shown). The continuous UNGD activity metric did not differ significantly (*p* = 0.26) between study participants and questionnaire nonresponders (Table 1). Study participants were less likely than nonresponders to be in the highest UNGD quartile. Although the Charlson comorbidity index was higher in responders (mean = 3.43) than in nonresponders (mean = 2.52, *p* < 0.001), the mean Charlson values were similar across all UNGD quartiles (Table 1).

**Table 1:** Comparison of selected characteristics in survey responders and nonresponders.

Characteristic	Responders (*n* = 7,785)	Nonresponders (*n* = 15,525)	*p*-Value
Continuous composite UNGD activity metric, mean ± sd	–0.02 ± 1.80	0.01 ± 2.78	0.26^*a*^
UNGD activity, *n* (%)
Quartile 1	2,052 (26.4)	3,775 (24.3)	< 0.001^*b*^
Quartile 2	1,828 (23.5)	3,996 (25.7)
Quartile 3	2,017 (25.9)	3,814 (24.6)
Quartile 4	1,888 (24.3)	3,940 (25.4)
Charlson index, mean ± sd	3.43 ± 2.76	2.52 ± 2.65	< 0.001^*a*^
Charlson index stratified by quartiles of UNGD activity, mean ± sd
Quartile 1	3.27 ± 2.61	2.46 ± 2.46
Quartile 2	3.37 ± 2.71	2.48 ± 2.57	NA
Quartile 3	3.61 ± 2.83	2.68 ± 2.85
Quartile 4	3.47 ± 2.86	2.48 ± 2.70
	*p* < 0.001^*c*^	*p* < 0.001^*c*^
Notes: NA, not applicable; sd, standard deviation; UNGD, unconventional natural gas development. Patients who lived outside Pennsylvania were excluded (*n* = 390). UNGD activity was averaged over 90 days before the survey. ^***a***^*p*-Value computed using Student’s *t*-test. ^***b***^*p*-Value computed using Chi-squared test. ^***c***^Within responders and nonresponders separately, *p*-values were computed with one-way analysis of variance (ANOVA) to compare mean Charlson index across quartiles of UNGD.			

We identified 738 participants with current CRS and no other primary outcome, 580 with migraine headache only, and 666 with higher levels of fatigue only (Table 2). These conditions were co-occurring in other individuals. There were 268 individuals with CRS and migraine, 347 with CRS and higher levels of fatigue, 420 with migraine and higher levels of fatigue, and 497 with all three outcomes. There were 1,380 participants with no current or past CRS, no migraine headache symptoms, and lower levels of fatigue; these individuals comprised the reference group. Compared with the reference group, individuals with each single outcome were more likely to be younger and current smokers (Table 2). Those with migraine and fatigue were more likely to be female, and those reporting CRS and fatigue were more likely to be white non-Hispanic.

**Table 2:** Characteristics of study population by self-reported outcome(s).

Characteristic	Overall study population	Individuals with no primary outcome		Individuals with one or more primary outcomes						
		Reference group^*a*^	Individuals who were neither cases nor controls^*b*^	Current CRS only	Migraine headache only	Higher levels of fatigue only	Current CRS and migraine	Current CRS and higher levels of fatigue	Migraine and higher levels of fatigue	Current CRS, migraine headache, and higher levels of fatigue
Total number, *n*	7,785	1,380	2,889	738	580	666	268	347	420	497
Sex, *n* (%)
Male	2,909 (37.4)	656 (47.5)	1,242 (43.0)	335 (45.4)	113 (19.5)	233 (35.0)	50 (18.7)	126 (36.3)	63 (15.0)	91 (18.3)
Female	4,876 (62.6)	724 (52.5)	1,647 (57.0)	403 (54.6)	467 (80.5)	433 (65.0)	218 (81.3)	221 (63.7)	357 (85.0)	406 (81.7)
Race/ethnicity, *n* (%)
White non-Hispanic	7,043 (90.5)	1,183 (85.7)	2,653 (91.8)	707 (95.8)	508 (87.6)	598 (89.8)	257 (95.9)	333 (96.0)	357 (85.0)	447 (89.9)
Other	742 (9.5)	197 (14.3)	236 (8.2)	31 (4.2)	72 (12.4)	68 (10.2)	11 (4.1)	14 (4.0)	63 (15.0)	50 (10.1)
Age in years, mean ± sd	55.3 ± 16.1	58.8 ± 17.0	57.6 ± 15.9	57.1 ± 14.9	46.1 ± 14.3	57.3 ± 15.1	48.5 ± 13.2	56.1 ± 14.7	46.5 ± 13.6	47.8 ± 13.1
Smoking status, *n* (%)
Never	4,268 (54.8)	805 (58.3)	1,615 (55.9)	404 (54.7)	340 (58.6)	334 (50.2)	141 (52.6)	178 (51.3)	220 (52.4)	231 (46.5)
Current	1,130 (14.5)	134 (9.7)	353 (12.2)	100 (13.6)	96 (16.6)	113 (17.0)	57 (21.3)	61 (17.6)	86 (20.5)	130 (26.2)
Former	2,387 (30.7)	441 (32.0)	921 (31.9)	234 (31.7)	144 (24.8)	219 (32.9)	70 (26.1)	108 (31.1)	114 (27.1)	136 (27.4)
History of receiving medical assistance, *n* (%)
Never	6,876 (88.3)	1,286 (93.2)	2,690 (93.1)	694 (94.0)	467 (80.5)	588 (88.3)	216 (80.6)	293 (84.4)	302 (71.9)	340 (68.4)
Ever	909 (11.7)	94 (6.8)	199 (6.9)	44 (6.0)	113 (19.5)	78 (11.7)	52 (19.4)	54 (15.6)	118 (28.1)	157 (31.6)
Body mass index (kg/m^2^), mean ± sd	30.2 ± 7.0	29.0 ± 6.3	29.9 ± 6.5	30.4 ± 7.0	29.7 ± 7.3	31.7 ± 7.9	29.8 ± 7.3	31.3 ± 7.4	31.7 ± 7.7	31.2 ± 8.1
Place type, *n* (%)
Township	4,949 (63.6)	907 (65.7)	1,900 (65.8)	476 (64.5)	332 (57.2)	417 (62.6)	170 (63.4)	213 (61.4)	242 (57.6)	292 (58.8)
Borough	2,135 (27.4)	371 (26.9)	762 (26.4)	188 (25.5)	183 (31.6)	192 (28.8)	72 (26.9)	101 (29.1)	122 (29.0)	144 (29.0)
Census tract in city	701 (9.0)	102 (7.4)	227 (7.9)	74 (10.0)	65 (11.2)	57 (8.6)	26 (9.7)	33 (9.5)	56 (13.3)	61 (12.3)
Community socioeconomic deprivation, mean ± sd	0.0 ± 3.6	–0.3 ± 3.6	–0.1 ± 3.6	–0.1 ± 3.5	0.3 ± 3.7	0.1 ± 3.6	0.2 ± 3.5	0.1 ± 3.7	0.6 ± 3.7	0.6 ± 3.8
UNGD activity metric, *n* (%)^*c*^
Quartile 1 [–0.61 to –0.47]	1,946 (25.0)	358 (25.9)	745 (25.8)	181 (24.5)	140 (24.1)	155 (23.3)	63 (23.5)	91 (26.2)	101 (24.0)	112 (22.5)
Quartile 2 [–0.47 to –0.39]	1,946 (25.0)	345 (25.0)	731 (25.3)	187 (25.3)	145 (25.0)	174 (26.1)	65 (24.3)	83 (23.9)	92 (21.9)	124 (24.9)
Quartile 3 [–0.39 to –0.16]	1,946 (25.0)	373 (27.0)	733 (25.4)	188 (25.5)	131 (22.6)	172 (25.8)	70 (26.1)	73 (21.0)	98 (23.3)	108 (21.7)
Quartile 4 [> –0.16]	1,947 (25.0)	304 (22.0)	680 (23.5)	182 (24.7)	164 (28.3)	165 (24.8)	70 (26.1)	100 (28.8)	129 (30.7)	153 (30.8)
Notes: CRS, chronic rhinosinusitis; sd, standard deviation; UNGD, unconventional natural gas development. Percentages may not total 100 because of rounding. ^***a***^Individuals in the reference group reported no past or current CRS; no headache-related nausea, photophobia, or disability; and lower levels (≤ 25th percentile) of fatigue. ^***b***^These individuals did not meet criteria for any primary outcome and were excluded from the reference group because of past CRS, intermediate probability of migraine headache, moderate levels of fatigue, or a combination of any of these symptoms. ^***c***^UNGD activity was averaged over the 90 days before the survey.										

### Associations of UNGD with Symptoms

**Figure 2:**
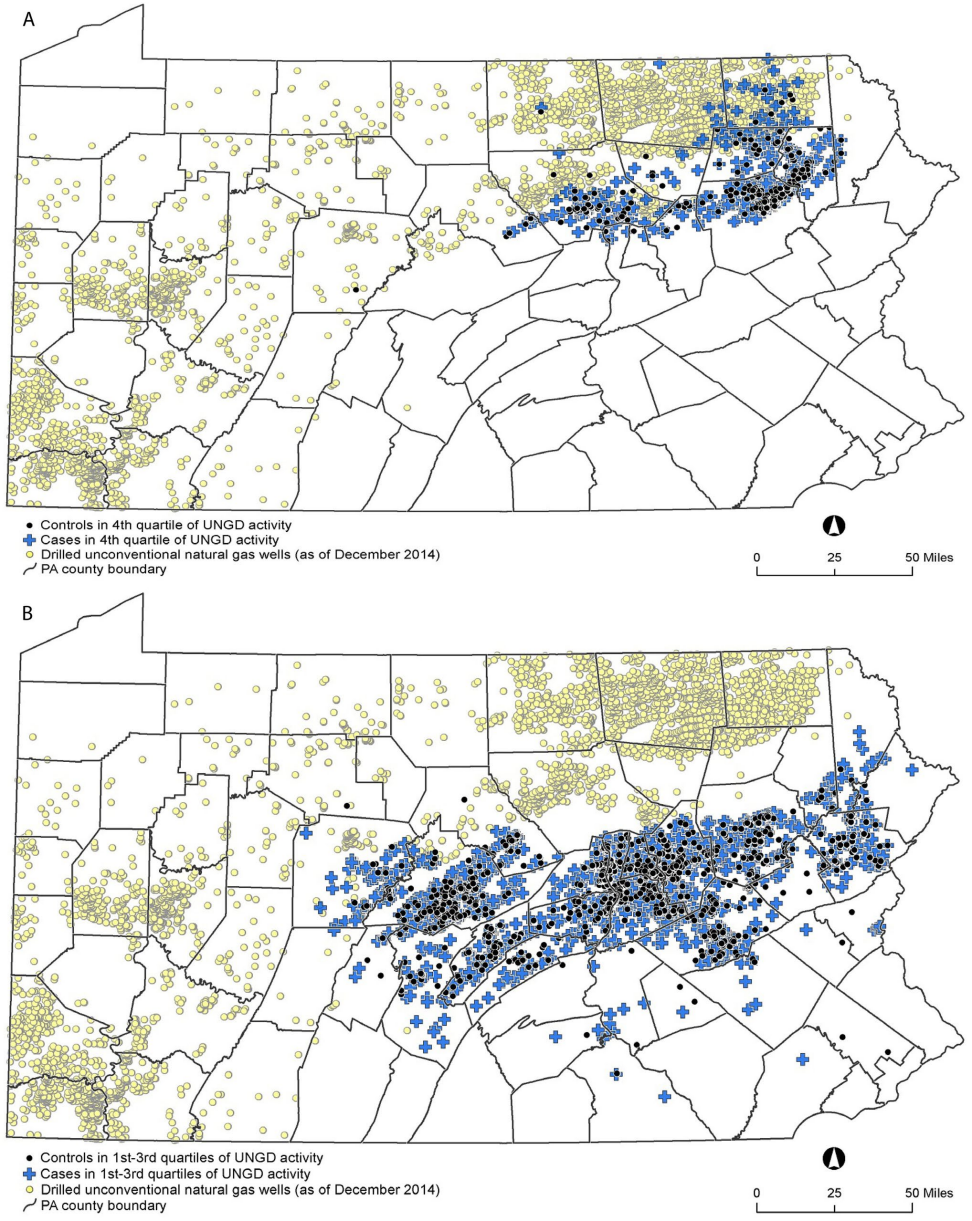
Locations of study participants in the fourth quartile of unconventional natural gas develpment (UNGD) activity (*A*) and all other UNGD quartiles (*B*). Blue crosses: participants with at least one primary outcome [current chronic rhinosinusitis (CRS), migraine headache, and/or higher levels of fatigue]. Black circles: reference group participants with no current or past CRS, no migraine headache symptoms, and lower levels of fatigue. Yellow circles: locations of all drilled unconventional natural gas wells in Pennsylvania as of 31 December 2014. Patient residential locations were from the Geisinger Clinic; county boundaries from the U.S. Census Bureau TIGER/line files (U.S. Census Bureau 2010); and UNGD well locations from the Pennsylvania Department of Environmental Protection (Pennsylvania Department of Environmental Protection 2016). Maps were made with ArcGIS Desktop (release 10, Esri, Redlands, CA).

The highest quartile of UNGD activity, compared with the lowest, was associated with significantly increased odds of the following combinations of two or more outcomes: CRS and higher levels of fatigue [odds ratio (OR) = 1.88; 95% confidence interval (CI): 1.08, 3.25], migraine headache and higher levels of fatigue (OR = 1.95; 95% CI: 1.18, 3.21), and all three outcomes (OR = 1.84; 95% CI: 1.08, 3.14) (Table 3). The second and third quartiles of UNGD were not significantly associated with any of the outcomes. In individuals with only one outcome, the odds ratios for the fourth quartile of UNGD were 1.11 (95% CI: 0.75, 1.65) for current CRS, 1.43 (95% CI: 0.94, 2.18) for migraine headache, and 1.47 (95% CI: 0.996, 2.18) for higher levels of fatigue (Table 3). In general, participants in the fourth quartile of UNGD lived farther north than those in other UNGD quartiles (Figure 2).

**Table 3:** Associations of UNGD with symptoms in individuals with one or more primary outcomes, compared with a reference group.

UNGD quartile	Adjusted odds ratios (95% confidence intervals)						
	Current CRS only (*n *= 736)^*a*^	Migraine headache only (*n *= 580)	Higher levels of fatigue only (*n *= 666)	Current CRS and migraine (*n *= 266)^*a*^	Current CRS and higher levels of fatigue (*n *= 347)^*a*^	Migraine and higher levels of fatigue (*n *= 420)	All three outcomes (*n *= 496)^*a*^
1	1.00 (reference)	1.00 (reference)	1.00 (reference)	1.00 (reference)	1.00 (reference)	1.00 (reference)	1.00 (reference)
2	1.17 (0.80, 1.72)	1.14 (0.74, 1.75)	1.48 (1.01, 2.17)	0.82 (0.43, 1.57)	1.06 (0.62, 1.80)	1.06 (0.63, 1.78)	1.05 (0.63, 1.78)
3	0.76 (0.52, 1.12)	0.89 (0.58, 1.36)	1.22 (0.84, 1.77)	0.74 (0.38, 1.47)	0.94 (0.53, 1.66)	0.80 (0.49, 1.31)	0.73 (0.42, 1.27)
4	1.11 (0.75, 1.65)	1.43 (0.94, 2.18)	1.47 (0.996, 2.18)	1.49 (0.78, 2.85)	1.88 (1.08, 3.25)	1.95 (1.18, 3.21)	1.84 (1.08, 3.14)
Notes: CRS, chronic rhinosinusitis; UNGD, unconventional natural gas development. For all models, the reference group consisted of individuals with no current or past CRS, no migraine headache symptoms, and the lowest quartile of fatigue score. All models included sampling weights, with the highest weight truncated to the value of the second-highest weight. Models included the following covariates: sex, race/ethnicity (white non-Hispanic vs. other), centered age (linear and quadratic terms), Medical Assistance (never vs. ever), and smoking status (never vs. current and former). UNGD activity was averaged over the 90 days before the survey. ^***a***^These models included centered body mass index as an additional covariate. Because individuals with unknown body mass index were excluded, these case counts are slightly lower than those reported in the text.							

When we stratified CRS patients by onset date, the second (OR = 3.27; 95% CI: 1.21, 8.82) and fourth (OR = 3.26; 95% CI: 1.14, 9.36) quartiles of UNGD were associated with significantly increased odds of CRS in those whose symptoms began after 2006 (see Table S2). There were no associations in participants with earlier symptom onset.

### Sensitivity Analyses

In participants with multiple outcomes, most inferences were unchanged whether we used the full sampling weights, truncated weights, or no weights (compare Table 3 with Table S3). Odds ratios for the fourth quartile of UNGD were consistently higher, and had wider confidence intervals, in fully weighted models than in models with truncated weights. For example, the odds ratio for the association of the fourth quartile of UNGD with the coexistence of migraine and fatigue was 2.89 (95% CI: 1.45, 5.76) in the fully weighted model. In individuals with single outcomes, the fourth quartile of UNGD was significantly associated with migraine headache (OR = 1.80; 95% CI: 1.02, 3.17) and fatigue (OR = 1.89; 95% CI: 1.10, 3.26) in the models with full weights; significant associations were also present in unweighted models (see Table S3).

UNGD activity, when averaged over 7 or 365 days, was highly correlated with the 90-day time-averaged UNGD metric used in the primary analyses (Spearman coefficient = 0.98 for both comparisons). Most inferences and associations were similar when using a 7-day or 365-day averaging period (see Table S4). The second quartile of UNGD was associated with past CRS, but there were no associations of UNGD with moderate levels of fatigue (see Table S5). UNGD was not associated with the negative control outcomes of ear pain, bad breath, or cold/flu symptoms (Table 4).

**Table 4:** Associations of UNGD with negative control outcomes.

UNGD quartile	Adjusted odds ratios (95% confidence intervals)		
	Ear pain yes (*n *= 422) vs. no (*n *= 3,917)	Bad breath yes (*n *= 846) vs. no (*n *= 2,628)	Cold/flu symptoms yes (*n *= 307) vs. no (*n *= 2,442)
1	1.00 (reference)	1.00 (reference)	1.00 (reference)
2	0.92 (0.58, 1.44)	0.87 (0.61, 1.22)	1.04 (0.58, 1.84)
3	0.53 (0.32, 0.87)	1.12 (0.80, 1.57)	1.15 (0.66, 2.00)
4	1.16 (0.74, 1.83)	0.95 (0.67, 1.35)	1.14 (0.64, 2.01)
UNGD, unconventional natural gas development. Individuals having the symptom at least “most of the time” in the past 3 months were compared with those having the symptom “never” in the past 3 months. All models included sampling and response weights, and the highest weight was truncated to the value of the second-highest weight. Models included the following covariates: sex, race/ethnicity (white non-Hispanic vs. other), centered age (linear and quadratic terms), Medical Assistance (never vs. ever), and smoking status (never vs. current and former). UNGD activity was averaged over the 90 days before the survey.			

Because only the highest level of UNGD was associated with our primary outcomes, we compared demographic and socioeconomic characteristics of individuals in the fourth quartile of UNGD with those of participants in other UNGD quartiles (see Table S6). Participants in the fourth quartile of UNGD differed on some covariates, several of which were included in the final models. We did not include place type in the final adjusted models because it could be a surrogate for mediators (e.g., individual- or place-level socioeconomic status) of associations between UNGD and symptoms. In a sensitivity analysis that explored the effect of place type, some associations were slightly attenuated when place type was added to the models, but inferences were similar (see Table S7).

## Discussion

In our survey of primary care patients in central and northeast Pennsylvania, residential UNGD activity was associated with nasal and sinus symptoms, migraine headache, and higher levels of fatigue, either alone or in combination. Our findings are suggestive of a threshold in the relationship between UNGD and symptoms because associations were present only among participants in the fourth quartile of UNGD activity. We found stronger associations in individuals with two or more co-occurring outcomes. In addition, UNGD was associated with CRS in individuals whose nasal and sinus symptoms began after the start of UNGD in Pennsylvania, although these estimates had lower precision owing to the small number of subjects with recent CRS onset.

In surveys such as ours, in which selection is based on the outcome, regression models must include sampling weights (or employ another strategy to acknowledge the selection mechanism) to avoid bias. However, extreme sampling weights can significantly increase the model’s variance (Potter 1988). To balance bias reduction against variance inflation, several techniques have been developed to truncate large sampling weights. We employed one such technique in our primary analyses. We found associations between UNGD and symptoms in the primary models as well as in fully weighted and unweighted models.

There is limited prior evidence linking environmental factors to CRS, migraine headache, and fatigue. Exposure to allergens, toxicants, and secondhand smoke may trigger nasal and sinus symptoms (Fokkens et al. 2012). However, a recent review found insufficient epidemiologic evidence from which to draw conclusions about occupational or environmental risk factors for CRS (Sundaresan et al. 2015). Although migraines have a strong hormonal and genetic component, they can also be triggered by noise, odors, and stress (Friedman and De ver Dye 2009; Sjöstrand et al. 2010; Sauro and Becker 2009). Similarly, fatigue has multiple risk factors including sleep deprivation, psychosocial stressors, medical disorders, psychiatric factors, occupation, and exposure to low levels of environmental chemicals (Bell et al. 1998; Ranjith 2005; Ricci et al. 2007; Griffith and Zarrouf 2008). Our UNGD activity metrics were designed to capture all potential environmental pathways that could affect these symptoms.

We did not measure participants’ exposure to ambient air pollution. We also did not account for conventional oil and gas wells. During our study period, the production of conventional gas wells in Pennsylvania was very low compared with that of unconventional wells. Furthermore, Pennsylvania’s conventional wells tend to be in the northwest and west, where Geisinger has no clinics/hospitals. The lack of significant geographic overlap with our study population makes confounding of UNGD associations by conventional oil and gas wells unlikely.

Participants in the fourth quartile of UNGD activity lived farther north than those in other quartiles (Figure 2). This spatial separation is due to the location of the Marcellus shale, which constrains UNGD to the northern portion of the Geisinger catchment area. Given the correlation between geography and UNGD, we cannot rule out the possibility that spatial confounding was responsible for the observed associations. However, we note that our models were adjusted for several covariates (such as race/ethnicity and socioeconomic status) that could be associated with both location and outcome. In addition, the null results in our negative control outcome models did not suggest spatial confounding.

CRS, migraine headache, and fatigue are highly prevalent and produce significant societal costs. CRS affects 2–16% of U.S. adults and results in emergency department visits, antibiotic prescriptions, sinus surgeries, and direct healthcare costs (Hastan et al. 2011; Bhattacharyya 2009; Shashy et al. 2004; Tan et al. 2013). Migraines have a prevalence of 11–14% and cause substantial temporary disability, emergency department visits, outpatient clinic visits, and analgesic use (Lipton et al. 2007; Burch et al. 2015). Fatigue prevalence, defined in various ways across studies, is estimated at 7–45%, and fatigue costs U.S. employers > 100 billion USD per year in lost productive work time (Ricci et al. 2007). From a public health and economic perspective, it is vital to understand modifiable risk factors for these illnesses.

Recent reviews have noted the lack of high-quality evidence regarding the health effects of UNGD (Adgate et al. 2014; Werner et al. 2015). Our study of 7,785 Pennsylvania residents is the largest survey of symptoms with respect to UNGD and has several strengths when compared with prior studies. We selected a population-based adult sample with no exclusion criteria. Reporting bias was minimized by the fact that UNGD was not identified as a study aim, and response rates did not differ by proximity to UNGD. Our time-varying UNGD activity metric incorporated well phase and intensity measures such as total depth and gas production. We used standardized and validated instruments to assess fatigue and migraine, respectively, and we used consensus epidemiologic guidelines to assess CRS.

This study had several limitations. In general, cross-sectional surveys such as ours cannot assess temporal relationships between exposures and outcomes, and we did not ascertain the onset dates of some symptoms. We note, however, that our UNGD activity metrics could theoretically be used to establish temporality because they can be computed for any date prior to symptom onset. Our ascertainment of self-reported outcomes was susceptible to various types of information bias. For example, despite the fact that our questionnaire did not mention UNGD, individuals residing near UNGD may have over-reported symptoms. There was some evidence of selection bias because survey participants had poorer health (measured by the Charlson comorbidity index) than nonresponders. However, differences in health status were similar across levels of UNGD activity. Another limitation is that our estimates of well development phase durations, although based on published average values, may have been incorrect for individual wells. Further exposure misclassification could have occurred because our UNGD activity metric was based on residential addresses. Participants’ exposure to UNGD activity could have been affected by unmeasured factors such as occupation, travel, and time spent outdoors. Additionally, our UNGD activity metric did not allow identification of specific exposures or exposure pathways.

## Conclusions

UNGD was associated with CRS, migraine headache, and fatigue symptoms in a large population-based survey. Associations were stronger in patients with two or more outcomes. Our work has several advantages over previous studies, making it an important addition to the growing body of evidence that UNGD is associated with adverse health effects. Further research, including more sophisticated exposure and outcome measurements, is necessary to evaluate whether these associations are causal and to elucidate the mechanisms for these findings.

## Supplemental Material

(356 KB) PDF

(299 KB) ZIP
