# Supplementary material for: Associations between Unconventional Natural Gas Development and Nasal and Sinus, Migraine Headache, and Fatigue Symptoms in Pennsylvania
Source: Environ Health Perspect. 2016 Aug 25;125(2):189–97. doi: 10.1289/EHP281 (PMC5289909; doi:10.1289/EHP281)
Supplement: (356 KB) PDF [file EHP281.s001.acco.pdf]

Environ Health Perspect
DOI: 10.1289/EHP281

Note to readers with disabilities: *EHP* strives to ensure that all journal content is accessible to all readers. However, some figures and Supplemental Material published in *EHP* articles may not conform to 508 standards due to the complexity of the information being presented. If you need assistance accessing journal content, please contact ehp508@niehs.nih.gov. Our staff will work with you to assess and meet your accessibility needs within 3 working days.

Supplemental Material

Associations between Unconventional Natural Gas Development and Nasal and Sinus, Migraine Headache, and Fatigue Symptoms in Pennsylvania

Aaron W. Tustin, Annemarie G. Hirsch, Sara G. Rasmussen, Joan A. Casey, Karen Bandeen-Roche, and Brian S. Schwartz

Table of Contents

Models of past disease and moderate symptoms
Table S1. Stratified survey design, response frequencies, and sampling weights
Table S2. Associations of UNGD with current CRS, stratified by date of symptom onset
Table S3. Sensitivity analysis of the effects of sampling weights on associations between UNGD and symptoms
Table S4. Sensitivity analysis of different time averaging periods for UNGD activity
Table S5. Associations of UNGD with past CRS and moderate levels of fatigue
Table S6. Selected characteristics of individuals in the fourth quartile of UNGD, compared to those in other quartiles
Table S7. Effect of place type (township, borough, or census tract in city) on associations of UNGD with symptoms

Additional File
Supplemental Code and Data ZIP File
Population Study of Nasal and Sinus Symptoms

Models of Past Disease and Moderate Symptoms

In our primary analysis, 2889 study participants were classified as neither cases nor controls. These individuals did not meet case criteria and were excluded from the reference group because of past CRS, intermediate likelihood of migraine headache, and/or moderate fatigue symptoms.

In a sensitivity analysis, we created two logistic regression models in which we re-classified some of these excluded individuals as cases. The purpose of this analysis was to determine whether UNGD was associated with less severe or past symptoms. In the first model ("past CRS"), the case group comprised all study participants who met criteria for past CRS (as defined in the main text) but who did not meet criteria for migraine headache or higher levels of fatigue (n = 1238). In the second model ("moderate fatigue"), cases were all individuals with moderate levels of fatigue (defined as the third quartile of fatigue; $19 \leq$ fatigue score < 28) but who did not meet criteria for current CRS or migraine headache (n = 1132). Both models compared cases to the reference group, described in the main text, of individuals with no past or current CRS, no migraine headache symptoms, and lower levels of fatigue (n = 1380). Both models were adjusted for sex, race/ethnicity (non-Hispanic white vs. other), age (linear and quadratic terms), receipt of Medical Assistance (never vs. ever), and smoking status (never vs. former and current). In addition, the past CRS model was adjusted for centered body mass index.

Table S1. Stratified survey design, response frequencies, and sampling weights.

Race/ethnicity	Lower likelihood of CRS	Intermediate likelihood of CRS	Higher likelihood of CRS
White, non-Hispanic			
Identified using EHR, n	131,366	47,892	13,132
Survey recipients, n	2,775	4,224	12,209
Survey respondents, n[a]	871	1,481	4,691
Sampling weight[b]	150.82	32.34	2.80
Other			
Identified using EHR, n	5,991	2,026	362
Survey recipients, n	2,283	1,869	340
Survey respondents, n[a]	322	350	70
Sampling weight[b]	18.61	5.79	5.17

Abbreviations: EHR, electronic health record; CRS, chronic rhinosinusitis.

Based on EHR data, we stratified the source population by race/ethnicity and prior probability of having CRS. We then oversampled racial/ethnic minorities and individuals more likely to have CRS.

[a] Excludes respondents with residential addresses outside Pennsylvania (n = 62).

[b] Sampling weights were calculated by dividing the total number of individuals identified in the stratum by the number of survey respondents.

Table S2. Associations of UNGD with current CRS, stratified by date of symptom onset.

**Onset date**	**UNGD quartile**	**Adjusted odds ratios (95% confidence intervals)**
Before January 1, 2006 (n = 519)	1	1.00 (reference)
	2	1.13 (0.72, 1.76)
	3	0.77 (0.50, 1.21)
	4	1.12 (0.71, 1.76)
After January 1, 2006 (n = 89)	1	1.00 (reference)
	2	3.27 (1.21, 8.82)
	3	1.62 (0.56, 4.64)
	4	3.26 (1.14, 9.36)

Abbreviations: UNGD, unconventional natural gas development; CRS, chronic rhinosinusitis.

Models compared individuals with current CRS (and no other primary outcome) to a reference group with no current or past CRS, no migraine headache symptoms, and lower levels of fatigue.

Models used sampling weights to account for the stratified survey design and response rates. The highest weight was truncated to the value of the second-highest weight. Models included these covariates: sex, race/ethnicity (white non-Hispanic vs. other), centered age (years), square of centered age, Medical Assistance (never vs. ever), smoking status (never vs. current and former), and centered body mass index. Individuals with unknown body mass index were excluded. Participants with current CRS were excluded from this analysis if their questionnaire responses did not provide enough information for us to determine whether their date of CRS onset occurred before or after January 1, 2006. UNGD activity was averaged over the 90 days prior to the survey.

Table S3. Sensitivity analysis of the effects of sampling weights on associations between UNGD and symptoms.

Sampling weight method	UNGD quartile	Current CRS only (n = 736)[a]	Migraine headache only (n = 580)	Higher levels of fatigue only (n = 666)	CRS and migraine (n = 266)[a]	CRS and fatigue[a] (n = 347)[a]	Migraine and fatigue (n = 420)	All three outcomes (n = 496)[a]
		Adjusted odds ratios (95% confidence intervals)						
Full weights	1	1.00 (reference)	1.00 (reference)	1.00 (reference)	1.00 (reference)	1.00 (reference)	1.00 (reference)	1.00 (reference)
	2	1.11 (0.66, 1.88)	1.12 (0.64, 1.97)	1.62 (0.96, 2.72)	0.81 (0.37, 1.79)	1.45 (0.69, 3.06)	1.44 (0.73, 2.84)	1.75 (0.83, 3.69)
	3	0.65 (0.38, 1.13)	0.88 (0.49, 1.57)	1.33 (0.79, 2.22)	0.47 (0.21, 1.09)	1.43 (0.65, 3.16)	0.84 (0.42, 1.67)	0.60 (0.29, 1.23)
	4	1.02 (0.59, 1.76)	1.80 (1.02, 3.17)	1.89 (1.10, 3.26)	1.91 (0.75, 4.82)	2.50 (1.16, 5.41)	2.89 (1.45, 5.76)	2.58 (1.20, 5.54)
Unweighted	1	1.00 (reference)	1.00 (reference)	1.00 (reference)	1.00 (reference)	1.00 (reference)	1.00 (reference)	1.00 (reference)
	2	1.22 (0.94, 1.58)	1.15 (0.85, 1.57)	1.21 (0.92, 1.59)	1.19 (0.78, 1.80)	1.02 (0.72, 1.45)	1.01 (0.70, 1.46)	1.17 (0.83, 1.65)
	3	1.03 (0.80, 1.34)	0.85 (0.63, 1.17)	1.06 (0.81, 1.38)	0.95 (0.63, 1.44)	0.79 (0.55, 1.13)	0.86 (0.60, 1.24)	0.83 (0.58, 1.18)
	4	1.30 (0.998, 1.70)	1.46 (1.07, 2.00)	1.32 (1.01, 1.74)	1.28 (0.84, 1.95)	1.38 (0.99, 1.95)	1.64 (1.15, 2.34)	1.73 (1.23, 2.44)

Abbreviations: UNGD, unconventional natural gas development; CRS, chronic rhinosinusitis.

Each model compared individuals with symptoms to a reference group with no current or past CRS, no migraine headache symptoms, and lower levels of fatigue. Models included these covariates: sex, race/ethnicity (white non-Hispanic vs. other), centered age (years), square of centered age, Medical Assistance (never vs. ever), and smoking status (never vs. current and former). UNGD activity was averaged over the 90 days prior to the survey.

[a] These models also included centered body mass index. Because individuals with unknown body mass index were excluded, these case counts are slightly lower than those reported in the main text.

Table S4. Sensitivity analysis of different time averaging periods for UNGD activity.

Outcome(s)	UNGD quartile	7-day averaged UNGD metric	365-day averaged UNGD metric
		Adjusted odds ratios (95% confidence intervals)	
CRS symptoms only (n = 736)[a]	1	1.00 (reference)	1.00 (reference)
	2	1.23 (0.84, 1.81)	1.38 (0.94, 2.03)
	3	0.76 (0.52, 1.13)	0.80 (0.54, 1.18)
	4	1.12 (0.75, 1.67)	1.16 (0.78, 1.71)
Migraine headache only (n = 580)	1	1.00 (reference)	1.00 (reference)
	2	1.03 (0.67, 1.58)	1.42 (0.93, 2.18)
	3	0.83 (0.54, 1.28)	1.09 (0.71, 1.65)
	4	1.44 (0.93, 2.23)	1.50 (0.98, 2.29)
Higher levels of fatigue only (n = 666)	1	1.00 (reference)	1.00 (reference)
	2	1.43 (0.97, 2.09)	1.75 (1.19, 2.56)
	3	1.31 (0.90, 1.89)	1.46 (0.998, 2.13)
	4	1.40 (0.95, 2.08)	1.55 (1.04, 2.30)
CRS and migraine (n = 266)[a]	1	1.00 (reference)	1.00 (reference)
	2	0.66 (0.35, 1.25)	1.05 (0.57, 1.96)
	3	0.73 (0.38, 1.41)	0.76 (0.38, 1.55)
	4	1.31 (0.69, 2.49)	1.64 (0.86, 3.09)
CRS and fatigue (n = 347)[a]	1	1.00 (reference)	1.00 (reference)
	2	1.25 (0.73, 2.16)	0.86 (0.50, 1.50)
	3	1.24 (0.71, 2.16)	1.46 (0.86, 2.47)
	4	1.72 (0.97, 3.04)	1.41 (0.81, 2.47)
Migraine and fatigue (n = 420)	1	1.00 (reference)	1.00 (reference)
	2	1.29 (0.78, 2.15)	1.56 (0.94, 2.60)
	3	0.92 (0.55, 1.54)	1.16 (0.70, 1.91)
	4	2.05 (1.22, 3.43)	1.94 (1.17, 3.22)
All three outcomes (n = 496)[a]	1	1.00 (reference)	1.00 (reference)
	2	1.26 (0.74, 2.14)	1.06 (0.63, 1.77)
	3	0.85 (0.49, 1.49)	0.80 (0.47, 1.36)
	4	2.02 (1.19, 3.43)	1.79 (1.06, 3.03)

Abbreviations: UNGD, unconventional natural gas development; CRS, chronic rhinosinusitis.

In all models, the reference group consisted of individuals with no current or past CRS, no migraine headache symptoms, and lower levels of fatigue. All models used sampling weights to account for the stratified survey design and response rates. The highest sampling weight was truncated to the value of the second-highest weight. All models included these covariates: sex, race/ethnicity (white non-Hispanic vs. other), centered age (years), square of centered age, Medical Assistance (never vs. ever), smoking status (never vs. current and former).

[a] These models included centered body mass index. Because individuals with missing body mass index were excluded, these case counts are slightly lower than those reported in the main text.

Table S5. Associations of UNGD with past CRS and moderate levels of fatigue.

UNGD quartile	Model 1: Past CRS	Model 2: Moderate fatigue
	Adjusted odds ratios (95% confidence intervals)	
1	1.00 (reference)	1.00 (reference)
2	1.39 (1.01, 1.91)	0.99 (0.73, 1.35)
3	1.10 (0.80, 1.50)	0.90 (0.67, 1.22)
4	1.27 (0.91, 1.77)	1.03 (0.75, 1.42)

Abbreviations: UNGD, unconventional natural gas development; CRS, chronic rhinosinusitis.

The reference group for both models consisted of individuals with no current or past CRS, no migraine headache symptoms, and lower levels of fatigue. Models used sampling weights to account for the stratified survey design and response rates. The highest sampling weight was truncated to the value of the second-highest weight. Both models included these covariates: sex, race/ethnicity (white non-Hispanic vs. other), centered age (years), square of centered age, Medical Assistance (never vs. ever), smoking status (never vs. current and former). In addition, the past CRS model included centered body mass index.

Table S6. Selected characteristics of individuals in the fourth quartile of UNGD, compared to those in other quartiles.

**Characteristic**	**Fourth quartile of UNGD**	**All others**	***p*-value**
Total number	1947	5838	NA
Sex, n (%)			
Male	722 (37.1)	2187 (37.5)	0.79
Female	1225 (62.9)	3651 (62.5)	
Race/ethnicity, n (%)			
White non-Hispanic	1735 (89.1)	5308 (90.9)	0.02
Other	212 (10.9)	530 (9.1)	
Age in years, mean (sd)	54.8 (16.7)	55.4 (15.9)	0.16
Smoking status, n (%)			
Never	918 (47.1)	3350 (57.4)	< 0.001
Current	351 (18.0)	779 (13.3)	
Former	678 (34.8)	1709 (29.3)	
History of receiving Medical Assistance, n (%)			
Never	1669 (85.7)	5207 (89.2)	0.79
Ever	278 (14.3)	631 (10.8)	
Place type, n (%)			
Township	831 (42.7)	4118 (70.5)	< 0.001
Borough	667 (34.3)	1468 (25.1)	
Census tract in city	449 (23.1)	252 (4.3)	
Community socioeconomic deprivation, mean (sd)	0.6 (3.6)	-0.2 (3.6)	< 0.001
Education level, n (%)			
Less than high school	712 (36.6)	2210 (37.9)	0.90
High school graduate	81 (4.2)	236 (4.0)	
Some college	518 (26.6)	1528 (26.2)	
Bachelor's degree or higher	608 (31.2)	1784 (30.6)	
Missing	28 (1.4)	80 (1.4)	

Abbreviations: UNGD, unconventional natural gas development; NA, not applicable; sd, standard deviation. UNGD activity was averaged over the 90 days prior to the survey.

Table S7. Effect of place type (township, borough, or census tract in city) on associations of UNGD with symptoms.

Outcome(s)	UNGD quartile	Model 1: Base model	Model 2: Base model plus place type
		Adjusted odds ratios (95% confidence intervals)	
CRS symptoms only (n = 736)	1	1.00 (reference)	1.00 (reference)
	2	1.17 (0.80, 1.72)	1.15 (0.78, 1.70)
	3	0.76 (0.52, 1.12)	0.75 (0.51, 1.11)
	4	1.11 (0.75, 1.65)	0.99 (0.65, 1.51)
Migraine headache only (n = 580)	1	1.00 (reference)	1.00 (reference)
	2	1.14 (0.74, 1.75)	1.12 (0.73, 1.73)
	3	0.89 (0.58, 1.36)	0.89 (0.59, 1.36)
	4	1.43 (0.94, 2.18)	1.22 (0.78, 1.91)
Higher levels of fatigue only (n = 666)	1	1.00 (reference)	1.00 (reference)
	2	1.48 (1.01, 2.17)	1.48 (1.006, 2.16)
	3	1.22 (0.84, 1.77)	1.21 (0.83, 1.77)
	4	1.47 (0.996, 2.18)	1.34 (0.89, 2.02)
CRS and migraine (n = 266)	1	1.00 (reference)	1.00 (reference)
	2	0.82 (0.43, 1.57)	0.82 (0.43, 1.56)
	3	0.74 (0.38, 1.47)	0.76 (0.39, 1.51)
	4	1.49 (0.78, 2.85)	1.50 (0.75, 3.01)
CRS and fatigue (n = 347)	1	1.00 (reference)	1.00 (reference)
	2	1.06 (0.62, 1.80)	1.04 (0.61, 1.79)
	3	0.94 (0.53, 1.66)	0.94 (0.54, 1.67)
	4	1.88 (1.08, 3.25)	1.77 (0.99, 3.19)
Migraine and fatigue (n = 420)	1	1.00 (reference)	1.00 (reference)
	2	1.06 (0.63, 1.78)	1.05 (0.63, 1.76)
	3	0.80 (0.49, 1.31)	0.80 (0.49, 1.31)
	4	1.95 (1.18, 3.21)	1.79 (1.06, 3.03)
All three outcomes (n = 496)	1	1.00 (reference)	1.00 (reference)
	2	1.05 (0.63, 1.78)	1.05 (0.62, 1.78)
	3	0.73 (0.42, 1.27)	0.76 (0.44, 1.31)
	4	1.84 (1.08, 3.14)	1.81 (1.04, 3.15)

Abbreviations: UNGD, unconventional natural gas development; CRS, chronic rhinosinusitis. Covariates in the base model were sex, race/ethnicity, centered age (linear and quadratic terms), smoking status, Medical Assistance. CRS models also included centered body mass index. The reference group for all models consisted of individuals with no current or past CRS, no migraine headache symptoms, and lower levels of fatigue. All models used sampling weights, and the highest weight was reduced to the value of the second-highest weight.

The baseline questionnaire, “Population Study of Nasal and Sinus Symptoms,” is a separate PDF file in the Supplemental Code and Data ZIP File.
