# Supplementary material for: Associations between Unconventional Natural Gas Development and Nasal and Sinus, Migraine Headache, and Fatigue Symptoms in Pennsylvania
Source: Environ Health Perspect. 2016 Aug 25;125(2):189–97. doi: 10.1289/EHP281 (PMC5289909; doi:10.1289/EHP281)
Supplement: (299 KB) ZIP [file EHP281.s002.acco.zip › Population Study of Nasal and Sinus Symptoms.pdf]

Study ID____________________

Population Study of Nasal and Sinus Symptoms		
**Please answer each question to the best of your ability. Please complete this survey in one sitting. It should take around 10 to 15 minutes to complete. Use a pen to answer your questions. Please return the survey using the included self-addressed stamped envelope. Thank you for your time!**		

In your lifetime, have you ever had ...			
1.	Blockage of your nasal passages (nasal congestion) that lasted 3 months?	☐ Yes	☐ No
2.	Nasal discharge (runny nose) that was non-clear, green or yellow in color that lasted 3 months?	☐ Yes	☐ No
3.	Post-nasal drip (mucus dripping from the back of the nose into your throat) that lasted 3 months?	☐ Yes	☐ No
4.	Loss of sense of smell that lasted three months?	☐ Yes	☐ No
5.	Facial pain that lasted three months?	☐ Yes	☐ No
6.	Facial pressure that lasted three months?	☐ Yes	☐ No

SKIP NOTICE: If you answered "no" to all six questions above (questions #1-6), **then skip to question #33.**

7.	My nasal and sinus symptoms are only a problem during some seasons.	☐ Yes	☐ No	
8.	My nasal and sinus symptoms happen when I do NOT have a cold or flu.	☐ Yes	☐ No	
9.	At what age did you start experiencing the nasal or sinus symptoms listed in questions 1 through 6 for more than 3 months at a time?	☐ 0-10<br>☐ 11-15<br>☐ 16-20<br>☐ 21-25	☐ 26-30<br>☐ 31-35<br>☐ 36-40<br>☐ 41-45	☐ 46-50<br>☐ 51-55<br>☐ 56-60<br>☐ Over 60

The questions below are about breathing and nasal problems you may have had in the past 3 months.

On average, how often in the past three months have you had ...		Never	Once in a while	Some of the time	Most of the time	All the time
10.	Blockage of your nasal passages (nasal congestion)?	☐	☐	☐	☐	☐
11.	Nasal discharge that was yellow or green in color?	☐	☐	☐	☐	☐
12.	Post-nasal drip?	☐	☐	☐	☐	☐
13.	Loss of sense of smell?	☐	☐	☐	☐	☐
14.	Facial pain?	☐	☐	☐	☐	☐
15.	Facial pressure?	☐	☐	☐	☐	☐

SKIP NOTICE: If you answered "never" or "once in a while" to all six questions #10-15, **then skip to question #33.**

Check the box that describes how often each problem below has happened in the past three months, on average.		Never	Once in a while	Some of the time	Most of the time	All the time
16.	Both of my nasal passages have blockage.	☐	☐	☐	☐	☐
17.	At least one of my nasal passages is completely blocked.	☐	☐	☐	☐	☐
18.	I have been very bothered by my blocked nasal passage(s).	☐	☐	☐	☐	☐
19.	I have a lot of nasal discharge.	☐	☐	☐	☐	☐
20.	I have to blow my nose more than 10 times a day because of my nasal discharge.	☐	☐	☐	☐	☐
21.	I have been very bothered by my nasal discharge.	☐	☐	☐	☐	☐
22.	I have been coughing after I eat or lie down	☐	☐	☐	☐	☐
23.	I have had mucus in my throat that felt like a lump or blockage.	☐	☐	☐	☐	☐
24.	I have been very bothered by my post-nasal drip.	☐	☐	☐	☐	☐

In the past 3 months, on average…		Never	Once in a while	Some of the time	Most of the time	All the time
25.	I have not been able to smell anything.	☐	☐	☐	☐	☐
26.	I have been very bothered by my loss of sense of smell.	☐	☐	☐	☐	☐
27.	On a scale of 0 to 10, my facial pain has been at least a 5.<br>(0 is no pain and 10 is worst possible pain.)	☐	☐	☐	☐	☐
28.	I have been very bothered by my facial pain.	☐	☐	☐	☐	☐
29.	My facial pressure has been severe.	☐	☐	☐	☐	☐
30.	I have been very bothered by my facial pressure.	☐	☐	☐	☐	☐
31.	Where have you had facial pain in the past 3 months?	☐ Section 1<br>☐ Section 2<br>☐ Section 3<br>☐ Section 4<br>☐ I have not had facial pain			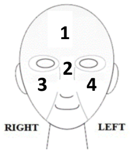 	
32.	Where have you had facial pressure in the past 3 months?	☐ Section 1<br>☐ Section 2<br>☐ Section 3<br>☐ Section 4<br>☐ I have not had facial pain			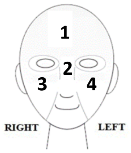 	

Check the box that describes how often, on average, you had the following problems in the past three months.

	SYMPTOM	Never	Once in a while	Some of the time	Most of the time	All the time
33.	Headaches	☐	☐	☐	☐	☐
34.	Fevers	☐	☐	☐	☐	☐
35.	Coughing	☐	☐	☐	☐	☐
36.	Bad breath	☐	☐	☐	☐	☐
37.	Fatigue	☐	☐	☐	☐	☐
38.	Nasal Itching	☐	☐	☐	☐	☐
39.	Sneezing	☐	☐	☐	☐	☐
40.	Eye Itching	☐	☐	☐	☐	☐
41.	Eye tearing	☐	☐	☐	☐	☐
42.	Ear fullness	☐	☐	☐	☐	☐
43.	Ear pain	☐	☐	☐	☐	☐
44.	Ear pressure	☐	☐	☐	☐	☐
45.	Wheezing (breathing with whistling sound in chest)	☐	☐	☐	☐	☐
46.	Chest tightness	☐	☐	☐	☐	☐
47.	Shortness of breath	☐	☐	☐	☐	☐
48.	Cold/ flu symptoms	☐	☐	☐	☐	☐

Check "YES" next to medications you have taken for sinus problems over the past three months. Include medications your doctor prescribed and over the counter medications. If you have not taken medication for sinus problems, check "NO."

49.	**Antibiotics** (Examples: Z-pack, Augmentin, Amoxicillin, Azithromycin, Doxycycline, Cipro, Levaquin)	☐ Yes	☐ No
50.	**Steroid pills** (Examples: Prednisone, Medrol dose pack, Methylprednisolone)	☐ Yes	☐ No
51.	**Nasal steroid sprays** (Examples: Nasonex, Mometasone, Flonase, Fluticasone)	☐ Yes	☐ No
52.	**Antihistamine pills** (Examples: Allegra, Claritin, Clarinase, Zyrtec)	☐ Yes	☐ No
53.	**Nasal antihistamines** (Examples: Astelin, Astepro, Azelastine, Patanase, Olopatidine)	☐ Yes	☐ No
54.	**Decongestants** (Examples: Sudafed, Afrin)	☐ Yes	☐ No

Please answer the questions below about sinus surgery.			
55.	How many times have you had sinus surgery?	☐ 0 – **SKIP to question 57.**<br>☐ 1	☐ 2<br>☐ 3 or more
56.	Why did you have sinus surgery? (Check all that apply)	☐ Chronic rhinosinusitis<br>☐ Other: ________________	☐ Nasal polyps<br>☐ Don't know

Check "yes" if a doctor has <u>ever</u> told you that you have any of these health problems.			
57.	Migraines	☐ Yes	☐ No
58.	Sinus problems	☐ Yes	☐ No
59.	Nasal polyps	☐ Yes	☐ No
60.	Asthma that is made worse by aspirin (aspirin intolerant asthma)	☐ Yes	☐ No
61.	Hay fever (seasonal allergies)	☐ Yes	☐ No
62.	Lupus	☐ Yes	☐ No
63.	Rheumatoid arthritis	☐ Yes	☐ No
64.	Psoriasis	☐ Yes	☐ No
65.	Inflammatory bowel disease	☐ Yes	☐ No
66.	Chronic rhinosinusitis (CRS) **(If NO, SKIP to question #68)**	☐ Yes	☐ No
67.	What test(s) did the doctor to do to test for CRS? (Check all that apply)	☐ CAT Scan<br>☐ Endoscopy<br>☐ MRI	☐ No test<br>☐ Don't know

Check "yes" if a doctor has <u>ever</u> told you that you have any of these health problems.			
68.	Nasal polyps **(If NO, SKIP to question #70)**	☐ Yes	☐ No
69.	What test(s) did the doctor to do to test for nasal polyps? (Check all that apply)	☐ CAT Scan<br>☐ Endoscopy<br>☐ MRI	☐ No test<br>☐ Don't know
70.	Have you ever had a blood test or skin test for allergies? **(If NO, SKIP to question #72)**	☐ Yes	☐ No
71.	Check the box next to the allergies that were found after a skin or blood test:	☐ Pollen<br>☐ Pets<br>☐ Mold<br>☐ Other:<br>__________	☐ Don't know<br>☐ No allergies

Place a check in the box that describes any wheezing or whistling in the chest you have experienced.

	Check the box that describes how often each problem below has happened in the <u>past twelve months</u>.	Never	Once in a while	Some of the time	Most of the time	All the time
72.	Wheezing, chest tightness, or whistling in the chest when you did not have a cold or the flu.	☐	☐	☐	☐	☐
73.	Woken at night due to wheezing.	☐	☐	☐	☐	☐
74.	Chest has sounded wheezy during or after exercise.	☐	☐	☐	☐	☐
75.	Dry cough at night, apart from a cough from a cold or chest infection.	☐	☐	☐	☐	☐

Answer the following questions about asthma.			
76.	Have you ever been told by a doctor that you have asthma?	☐ Yes	☐ No – **SKIP to question #80**
77.	Have you had your asthma symptoms in the last 12 months?	☐ Yes	☐ No
78.	How old were you when your asthma first began?	___ ___ years old	
79.	How old were you when you had your most recent asthma symptoms?	___ ___ years old	

Answer the following questions about headaches you have had in the past 12 months.			
80.	How often do you have headaches?	☐ Never<br>☐ Once in a while<br>☐ Some of the time	☐ Most of the time<br>☐ All of the time

SKIP NOTICE: If you checked "never" or "once in a while", **skip to question #84.**

		Never	Rarely	Less than half the time	Half the time or more
81.	How often do your headaches interfere with your ability to work, study, or enjoy life?	☐	☐	☐	☐
82.	How often do you have nausea with your headaches?	☐	☐	☐	☐
83.	How often have you been unusually sensitive to light during your headaches?	☐	☐	☐	☐

Please respond to each question or statement by marking one box per row.

	During the past 7 days...	Not at all	A little bit	Somewhat	Quite a bit	Very much
84.	I feel fatigued.	☐	☐	☐	☐	☐
85.	I have trouble starting things because I am tired.	☐	☐	☐	☐	☐
86.	How run-down did you feel on average?	☐	☐	☐	☐	☐
87.	How fatigued were you on average?	☐	☐	☐	☐	☐
88.	How much were you bothered by your fatigue on average?	☐	☐	☐	☐	☐
89.	To what degree did your fatigue interfere with your physical functioning?	☐	☐	☐	☐	☐
90.	How often did you have to push yourself to get things done because of your fatigue?	☐	☐	☐	☐	☐
91.	How often did you have trouble finishing things because of your fatigue?	☐	☐	☐	☐	☐

Please respond to the each question by marking one box per row.

92.	What is your current marital status?	☐ Married<br>☐ Separated<br>☐ Divorced	☐ Widowed<br>☐ Never Married<br>☐ Living with Partner	
93.	What is the highest grade of school or highest degree you have completed?	☐ No Schooling<br>☐ A few years<br>☐ Finished grammar school<br>☐ GED	☐ Some high school<br>☐ High school graduate<br>☐ Bachelor's degree	☐ Some college<br>☐ Associate degree<br>☐ Master's degree<br>☐ Doctoral degree
94.	About how much income did you receive last year before taxes and deductions?	☐ 0-9,999<br>☐ 10,000-24,999<br>☐ 25,000-49,999<br>☐ 50,000-74,999	☐ 75,000-99,999<br>☐ 100,000-149,999<br>☐ 150,000+	
